# Bacterial Production and Control of Biogenic Amines in Asian Fermented Soybean Foods

**DOI:** 10.3390/foods8020085

**Published:** 2019-02-25

**Authors:** Jae-Hyung Mah, Young Kyoung Park, Young Hun Jin, Jun-Hee Lee, Han-Joon Hwang

**Affiliations:** Department of Food and Biotechnology, Korea University, 2511 Sejong-ro, Sejong 30019, Korea; eskimo@korea.ac.kr (Y.K.P.); younghoonjin3090@korea.ac.kr (Y.H.J.); bory92@korea.ac.kr (J.-H.L.); hjhwang@korea.ac.kr (H.-J.H.)

**Keywords:** food safety, biogenic amines, fermented soybean foods, intervention methods, control, starter culture, *Bacillus* spp.

## Abstract

Fermented soybean foods possess significant health-promoting effects and are consumed worldwide, especially within Asia, but less attention has been paid to the safety of the foods. Since fermented soybean foods contain abundant amino acids and biogenic amine-producing microorganisms, it is necessary to understand the presence of biogenic amines in the foods. The amounts of biogenic amines in most products have been reported to be within safe levels. Conversely, certain products contain vasoactive biogenic amines greater than toxic levels. Nonetheless, government legislation regulating biogenic amines in fermented soybean foods is not found throughout the world. Therefore, it is necessary to provide strategies to reduce biogenic amine formation in the foods. Alongside numerous existing intervention methods, the use of *Bacillus* starter cultures capable of degrading and/or incapable of producing biogenic amines has been proposed as a guaranteed way to reduce biogenic amines in fermented soybean foods, considering that *Bacillus* species have been known as fermenting microorganisms responsible for biogenic amine formation in the foods. Molecular genetic studies of *Bacillus* genes involved in the formation and degradation of biogenic amines would be helpful in selecting starter cultures. This review summarizes the presence and control strategies of biogenic amines in fermented soybean foods.

## 1. Introduction

Microbial fermentation is one of the oldest and most practical technologies used in food processing and preservation. However, fermentation of protein-rich raw materials such as fish, meat, and soybean commonly provides abundant precursor amino acids of biogenic amines. Even though most fermented foods have been found to be beneficial to human health, biogenic amines produced through fermentation and/or contamination of protein-rich raw materials by amino acid-decarboxylating microorganisms may cause intoxication symptoms in human unless they are detoxified by human intestinal amine oxidases, viz., detoxification system [1,2]. Thus, the presence of biogenic amines in fermented foods (and non-fermented foods as well) has become one of the most important food safety issues.

According to old documents, the cultivation and use of soybeans, dating back to B.C., were launched in Manchuria on the north side of the Korean Peninsula and have spread to other regions of the world. Hence, a variety of fermented soybean foods have been developed and consumed in north-east Asian countries around the Korean Peninsula, and consequently humans in this region have steadily taken the fermented foods for a long period of time from hundreds to thousands of years, depending on the types of fermented soybean foods consumed [3]. Presently, fermented soybean foods are of public interest and consumed more frequently even in western leading countries because the fermented foods, particularly fermented soybean pastes, not only have been believed by many people, but also have been scientifically proven by researchers to have health-promoting and -protective effects [4]. However, much less attention has been paid to the safety issues of fermented soybean foods [5].

Fermented soybean foods, including various types of fermented soybean pastes and soy sauces, are commonly made from whole soybeans containing abundant amino acids through microbial fermentation. If the fermenting (or sometimes contaminating) microorganisms are significantly capable of decarboxylating amino acids, the resultant fermented soybean foods may contain unignorable amounts of biogenic amines. Indeed, the presence of biogenic amines seems to be quite frequent and inevitable in fermented soybean foods. Therefore, the present review provides information on the presence, bacterial production, and control strategies of biogenic amines in fermented soybean foods, especially focusing on fermented soybean pastes usually considered as heathy foods.

## 2. A Brief on Biogenic Amines

**Figure 1:**
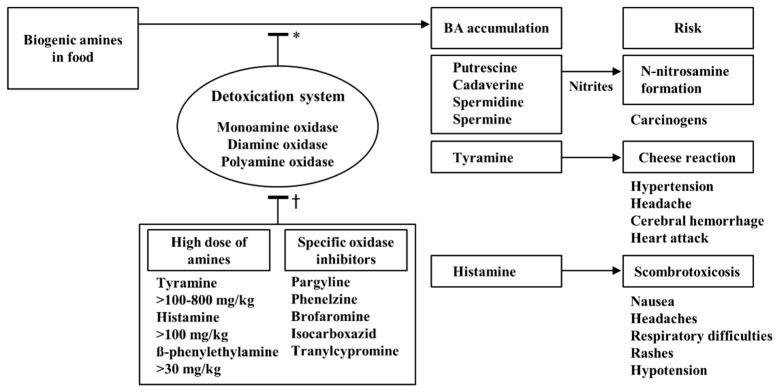
Detoxification and toxicological risks of biogenic amines. *: Metabolic inactivation of biogenic amines through oxidative deamination by oxidases. †: Incapacitation of intestinal detoxication system through saturation by biogenic amines or inhibition by antidepressant medications. BA: Biogenic amines.

Biogenic amines are defined as harmful nitrogenous compounds produced mainly by bacterial decarboxylation of amino acids in various foods. The bacterial decarboxylation of amino acids to biogenic amines have been well illustrated in literature and can be found elsewhere [6,7,8]. Biogenic amines are also endogenous and indispensable components of living cells, and consequently most food materials, including fruit, vegetables, and grains, contain different levels of biogenic amines depending on their variety, maturity and cultivation condition [7]. Usual intake of dietary biogenic amines generally causes no adverse reactions because human intestinal amine oxidases, such as monoamine oxidase (MAO), diamine oxidase (DAO) and polyamine oxidase (PAO), quickly metabolize and detoxify the biogenic amines. If the capacity of amine-metabolizing enzymes is over-saturated and/or the metabolic activity is impaired by specific inhibitors, vasoactive biogenic amines, including histamine, tyramine and β-phenylethylamine, may cause food intoxication and in turn be considered to be toxic substances in humans [1,2]. Furthermore, the toxicity of biogenic amines can be enhanced by putrefactive biogenic amines such as putrescine and cadaverine [9]. The most common symptoms of biogenic amine intoxication in human are nausea, respiratory distress, hot flushes, sweating, heart palpitation, headache, a bright red rash, oral burning, and hypo- or hypertension [10]. Figure 1 schematically illustrates the detoxification and toxicological risks of biogenic amines.

The biosynthesis, toxicity and physiological effects have been well reviewed in recent articles [11,12], and will not be summarized here. In addition, it is worth mentioning that in particular, vulnerable people who are immune compromised, such as the elderly, children, and infants, may exhibit intolerance to even low levels of biogenic amines and suffer more severe symptoms [13]. The maximum tolerance levels of vasoactive biogenic amines (mostly histamine in fish and fish products) have established and proposed by government agencies or individual researchers as described below (refer to Table 1), but may need to be further studies and subdivided, considering the vulnerable people.

## 3. Legal Limits and Toxic Levels of Biogenic Amines in Foods

Early in 1980, the U.S. Food and Drug Administration (FDA) first established regulations for tuna and mahimahi that consider 200 mg histamine/kg as an indication of prior mishandling and 500 mg histamine/kg as an indication of a potential health hazard [14]. Early in 1990, the European Economic Community (EEC) also established regulation for fish species of the *Scombridae* and *Clupeidae* families and fixed a three-class plan for maximum allowable levels of histamine in fresh fish (*n* = 9; *c* = 2; *m* = 100 ppm; *M* = 200 ppm) and enzymatically ripened fish products (*n* = 9; *c* = 2; *m* = 200 ppm; *M* = 400 ppm) where *n* is the number of units to be analyzed from each lot, *m* and *M* are the histamine tolerances, and *c* is the number of units allowed to contain a histamine level higher than *m* but lower than *M* [15]. In 1996, Shalaby [16] suggested the guidelines for histamine content of fish as follows: <50 mg/kg (safe for consumption), 50–200 mg/kg (possibly toxic), 200–1000 mg/kg (probably toxic), >1000 mg/kg (toxic and unsafe for human consumption) based on the review of the regulations and other literature. In the meantime, values of 100–800 mg/kg of tyramine and 30 mg/kg of β-phenylethylamine were reported to be toxic doses in food, respectively, and 100 mg histamine per kg of food and 2 mg histamine per liter of alcoholic beverage were suggested as upper limits for human consumption [6]. The upper limits and toxic doses (stated right above) suggested by Brink et al. [6] have been steadily used by numerous investigators as threshold values to assess human health risks derived from exposure to vasoactive biogenic amines in foods because there have been no other reports describing the guidelines for respective vasoactive biogenic amines in general foods, except for histamine (particularly in fish; not applicable to other foods).

At present, histamine is the only biogenic amine for which the U.S. FDA has set a guidance level, i.e., 50 mg/kg of histamine in the edible portion of fish [17], whereas the European Commission (EC) has established regulatory limits of 100 mg/kg for histamine in fish species and 400 mg/kg for histamine in fish sauce produced by fermentation of fishery products [18]. In the meantime, the European Food Safety Authority [19] reported that although a dose of 50 mg histamine is the no-observed-adverse-effect level (NOAEL), healthy individuals do not experience symptoms unless they ingest a larger amount of histamine than NOAEL. Then, the Food and Agriculture Organization/World Health Organization [20] announced 200 mg histamine/kg as the maximum allowable level for consumption of fish and fish products. According to the Codex standard [21], 200 mg/kg of histamine in fish and fish products and 400 mg/kg of histamine in fish sauce are set as the hygiene and handling indicator levels in the corresponding products, respectively. In addition, the governments of several countries in Asia and Oceania have lately established regulatory limits for histamine in fish and fish products [22,23,24]. Legal limits and toxic levels set by government agencies or individual researchers for biogenic amines in food products are listed in Table 1. Although several food scientists have referred the suggestion of Brink et al. [6], as described above, there have not been government regulations on maximum allowable levels of biogenic amines, other than histamine, in history. Besides, any government legislation or guidelines on the contents of biogenic amines in fermented soybean foods are not found throughout the world.

## 4. Fermented Soybean Foods and Vasoactive Biogenic Amines

Fermented soybean foods have not only been commonly consumed as they are, but have also been frequently used in a variety of processed products, which make them become a necessity in the household in Asian cultures. Moreover, fermented soybean food products have recently gained popularity, crossing from Asian communities to mainstream markets, in many western countries due to the healthy functions of the foods [3,4]. Aside from soy sauces, the most popular fermented soybean foods produced mainly by bacterial fermentation (sometimes with molds) are Natto, Miso (Japanese fermented soybean pastes), Cheonggukjang, Doenjang, Gochujang (Korean fermented soybean pastes), Chunjang, Doubanjiang, Douchi (Chinese fermented soybean pastes) and Tempeh (an Indonesian fermented soybean paste). Some other soybean foods such as Sufu (a Chinese fermented Tofu) and Tauco (an Indonesian fermented yellow soybeans) prepared by mold fermentation are also available in local area (but were excluded from this review due to great differences in the microorganisms involved in fermentation processes as well as little data available in literature). The safety issues of traditional fermented soybean foods have heretofore been overlooked because humans have consistently taken the foods at least for centuries or millennia. However, considering that fermented soybean foods contain not only abundant dietary amino acid precursors of biogenic amines, as mentioned at the beginning of this article, but also significant biogenic amine-producing microorganisms, mainly bacterial species, it is critically important to assess the levels of biogenic amines in the foods.

Based on a critical review of published data (refer to Table 2) [25,26,27,28,29,30,31,32,33,34,35,36,37], it seems that the amounts of biogenic amines in most fermented soybean food products are usually within the safe levels for human consumption. It is noteworthy, however, that some specimens of the fermented soybean food products, including both fermented soybean pastes and soy sauces, have been reported to contain vasoactive biogenic amines greater than toxic dose of each amine. For instance, β-phenylethylamine has been detected at concentrations up to 185.6 mg/kg and 239.0 mg/kg in Doubanjiang and Douchi, respectively [26,36], which are approximately 6-8 times higher than toxic dose of this amine (30 mg/kg) suggested by Brink et al. [6]. In another report, β-phenylethylamine was determined to be 8704.6 mg/kg in a Doenjang sample [34], but which is unreliably larger than those in other articles in which maximum β-phenylethylamine concentrations of 529.2 mg/kg and 544.0 mg/kg have been reported [28,32]; this report was thus excluded from further review. In the meantime, histamine has been detected at concentrations up to 952.0 mg/kg and 808.0 mg/kg in Doenjang and Douchi, respectively [28,36], whereas tyramine has been detected up to 1430.7 mg/kg and 2539.0 mg/kg in Doenjang and Cheonggukjang, respectively [28,33]. The maximum concentrations of histamine and tyramine reported are approximately 8–10 times higher than upper limit of histamine (100 mg/kg) and 14–25 times (on lower toxic dose basis; 2–3 times, upper dose basis) higher than toxic dose of tyramine (100–800 mg/kg), respectively, suggested by Brink et al. [6]. Like the fermented soybean pastes described above, some specimens of soy sauces have been reported to contain high levels of vasoactive biogenic amines, including β-phenylethylamine (up to maximum 121.6 mg/kg), histamine (398.8 mg/kg) and tyramine (794.3 mg/kg), which are much greater than toxic doses of respective amines [28]. As a counter-example, there is a report in which the amounts of respective vasoactive biogenic amines were very low or not detected in all samples (i.e., three batches) of commercial Natto, Miso, Tempeh, and soy sauce products; however, this report seems to insufficiently brief the presence of biogenic amines in the products because samples (batches) of only a single brand for each type of product were available in local stores [4]. The contents of biogenic amines in different types of fermented soybean food products reported in literature have been reviewed once in a book chapter in 2011 [38], and those in the representative fermented soybean food products reviewed herein are compiled in Table 2. After all, it seems likely that there may occasionally be a risk of food poisoning associated with eating fermented soybean pastes, especially when the pastes contain significant amounts of vasoactive biogenic amines, because some types of the pastes, for instance, Natto, Tempeh and sometimes Cheonggukjang, are taken not only as side dishes, but also main dishes. In the case of soy sauces, the risk to consumers may not be so great, considering the small quantity of intake per serve [29].

It is also worth pointing out that some specimens of fermented soybean food products have been found to contain relatively high levels of putrescine and cadaverine (Table 2). The putrefactive biogenic amines have been known to enhance the toxicity of vasoactive biogenic amines in foods [9]. Therefore, comprehensive monitoring and reduction strategies are required to reduce the risk of ingesting putrefactive biogenic amines as well as vasoactive biogenic amines in fermented soybean foods, which may come from the understanding of why there are differences in the amounts and diversity of biogenic amines between the types or batches of the food products. It is probably that the differences may be attributed to (i) the ratio of ingredients used in raw material, (ii) physicochemical and/or microbial contribution, and (iii) conditions and periods of the entire food supply chain [5]. Since fermented soybean foods have their own unique raw materials, physicochemical properties, and production processes, the present review focuses on bacterial contribution to biogenic amine formation conserved across most fermented soybean foods.

## 5. Bacterial Activity to Produce Biogenic Amines in Fermented Soybean Foods

It has been known that most fermented soybean foods, except for several types of soybean foods prepared by mold fermentation, are mainly fermented (or contaminated) by *Bacillus* species (particularly *B. subtilis*) [5,39,40], which, in turn, leads to biogenic amine formation in the fermented foods, although the abilities of *Bacillus* strains to produce biogenic amines are diverse depending on the types and/or batches of the food products from which the strains are isolated (refer to Table 3) [25,26,31,35,36,37]. In the studies, the reported ranges (mean ± standard deviation; minimum–maximum) of biogenic amines produced by *Bacillus* spp. in assay media, when cultured for 24 h with proper precursor amino acids, are as follows: histamine 0.22 ± 0.65–29.9 ± 13.4 μg/mL, tyramine 0.3 ± 0.5–30.6 ± 21.7 μg/mL, β-phenylethylamine not detected (ND)—11.2 ± 9.17 μg/mL, tryptamine 0.20 ± 0.45–6.17 ± 3.98 μg/mL, putrescine ND—7.59 ± 3.06 μg/mL, cadaverine ND—1.8 ± 1.1 μg/mL, spermidine 0.40 ± 0.20–9.26 ± 5.73 μg/mL, spermine 1.29 ± 0.86–27.2 ± 12.7 μg/mL. Among the *Bacillus* strains reported, *B. subtilis* strains isolated from Natto exhibited the strongest abilities to produce respective biogenic amines. Table 3 reveals the abilities to produce biogenic amines of different bacterial species isolated from representative types of fermented soybean food products.

In addition to the aforementioned *Bacillus* spp., *Lactobacillus* sp. and *Enterococcus faecium*, which had been isolated from raw materials of Miso, were proposed to produce histamine and tyramine in Miso, respectively, through a qualitative detection using BCP (Bromo-cresol purple) agar plates and subsequently a quantitative test using liquid media [41,42]. In the quantitative test with incubation for 90 days, the strains of *Lactobacillus* sp. and *E. faecium* produced histamine and tyramine up to approximately 100 μg/mL and 150 μg/mL, respectively. Although *Lactobacillus* species are not commonly involved in the preparation of fermented soybean foods, diverse species of *Lactobacillus* have also been reported to be responsible for the formation of biogenic amines, including histamine, in lactic fermented foods [12]. *E. faecium* and *E. faecalis* have been found to possess *tdc* gene and produce tyramine in fermented foods, including dairy products, fermented sausages, wine and fermented soybean foods [12]. Thus, *E. faecium* strains have been used as target organisms for studies on the reduction of tyramine in fermented soybean foods [33,43], even though *Enterococcus* spp. are present as contaminants at relatively low levels (maximum up to 10^6^ CFU/g) in the foods [44,45,46]. In the meantime, the absence of *hdc* gene encoding histidine decarboxylase was reported in both *E. faecium* and *E. faecalis* in one study [47], while histidine decarboxylase-positive *E. faecium* and *E. faecalis* strains were detected by a PCR (polymerase chain reaction) method in another study [48]. It is interesting to note that the PCR screening method used in the latter study employed the primers developed in the former study, which makes it difficult to conclude whether the species possess *hdc* gene or not.

As shown in Table 4, at present the Gene Bank database of the National Centre for Biotechnology Information (NCBI, National Center for Biotechnology Information, U.S. National Library of Medicine, Bethesda, MD, USA) provides the sequences of *tdc*, *odc*, and *ldc* genes in *E. faecium* and *tdc* and *ldc* genes in *E. faecalis*, while *hdc* gene sequence of both species is not available in the database. In contrast, the sequences of *hdc* gene in *B. licheniformis* and *B. coagulans* (this sequence is completely conserved between the two species) and *ldc* in *B. subtilis* have been deposited in the database, while *tdc* gene sequence is unavailable for the three species of *Bacillus*. Nevertheless, it has lately been suggested that *Bacillus* spp. are as significant as *Enterococcus* spp. for tyramine formation in fermented soybean foods [49]. The deposited genes encoding amino acid decarboxylases of *Bacillus* spp. and *Enterococcus* spp., the most important species related to biogenic amine formation in fermented soybean products, are listed in Table 4 (exceptionally, *odc*-Az encodes an antizyme inhibitor devoid of ornithine decarboxylase activity). All the bacteria and genes mentioned above should be targeted for preventive interventions to reduce biogenic amine formation in fermented soybean foods. Meanwhile, yeasts have been considered to produce only negligible amounts of biogenic amines [50,51]. Fungal distribution to biogenic amine accumulation is remained to be further studied because there appears to be but little literature available dealing with fungal formation of biogenic amines [52].

It is well known that various vasoactive and putrefactive biogenic amines are commonly formed by microbial decarboxylation of amino acids in fermented foods [6,7]. As such, it has been found that soybean fermentation results in an increase in the amount of spermine (and other biogenic amines), but a decrease in that of spermidine [26]. Since spermidine is essential for the growth and development of plants [53,54], this polyamine is abundantly present in soybean and non-fermented soybean foods such as Tofu (a curd product made from soy milk) [26,55,56] and degraded by bacterial enzymes during fermentation [57]. Consequently, fermented soybean foods contain a lower level of spermidine than their raw material, soybean [26,58]. This indicates that development and application of biogenic amine-degrading starter cultures are possible (and necessary) to reduce the contents of biogenic amines in fermented soybean foods. Identifying and understanding the dominant contributors to the formation of biogenic amines may facilitate the development of starter cultures for delaying or avoiding biogenic amine formation in the fermented foods. Taken together, it is clear that distinct and diverse bacterial community and/or capability of producing (and degrading) biogenic amines decisively determine the amounts and diversity of biogenic amines in fermented soybean foods.

## 6. Control Strategies for Reducing Biogenic Amines in Fermented Soybean Foods

Regarding intervention measures that reduce biogenic amine formation in fermented soybean foods, to date, only a few reports are available in literature as follows: the use of irradiation [59], addition of nicotinic acid as a tyrosine decarboxylase inhibitor [43], and use of *Bacillus* starter cultures [60,61,62,63]. However, when extended to other fermented foods, a review of the relevant literature reveals that several types of intervention methods have been developed and used to reduce biogenic amine contents in the foods (mainly fermented sausage and cheese), which involve chemical intervention, such as the use of food additives and natural antimicrobial compounds [43,64,65,66,67], physical intervention, such as the use of irradiation [59,68], high hydrostatic pressure [69,70] and modified atmosphere packaging [71,72], and biological intervention, particularly such as the use of starter cultures [60,61,62,63,73,74,75,76]. The biological intervention methods also involve the control or adjustment of intrinsic and extrinsic factors, such as alterations of temperature, pH, a_w_, and Eh, which have been well reviewed in literature [77,78,79].

Up to this day, thousands of additives have been used to extend shelf life of foods because of their antimicrobials, antioxidants, and antibrowning properties. Natural additives have lately been of great interest in food industry due to consumers’ health concerns [80]. Apart from being used as food preservatives, numerous food additives and natural antimicrobial compounds, including glycine [64], nicotinic acid [43], potassium sorbate, sodium benzoate [67], sodium chloride [64,66], clove [65,66,67], garlic [65], etc., have been found to be effective in suppressing bacterial ability to produce biogenic amines in foods. Among the compounds, nicotinic acid is only one compound proven to practically inhibit the formation of biogenic amines (particularly tyramine) in a fermented soybean food, viz., Cheonggukjang [43]. In the report, the addition of nicotinic acid at concentrations of 0.15% and 0.20% resulted in significant reductions, by approximately 70% and 83%, respectively, compared to the control, of tyramine content in the treated Cheonggukjang samples after 24 h of fermentation. In addition, it is worth noting that even though a successful reduction of biogenic amines in a food product can be achieved by the addition of any of a variety of compounds, some of the additives may cause organoleptic alterations, such as an atypical taste and flavor, in the final food product, especially in the case of fermented soybean foods [5,49]. Therefore, sensory evaluation should be incorporated as an integral part of a program investigating effective inhibitors of biogenic amine formation in fermented soybean foods.

Besides the chemical intervention measures described above, a variety of physical intervention processes have been developed and applied for food preservation, which involve not only well-known classical processes, for instance, heating, refrigeration, and freezing, but also emerging novel processes such as microwave heating, ohmic heating and pulsed electric fields developed during the past 25 to 35 years [81]. Among the physical intervention methods, irradiation, high hydrostatic pressure and modified atmosphere packaging have been relatively recently reported to successfully inhibit biogenic amine formation in fermented foods, which have been achieved mostly by reducing microbial population, for instance, lactic acid bacteria, closely related to the fermentation of foods [59,69,70,71,72]. Despite the technological progress that has been made, as for fermented soybean foods, there has been only a single report describing biogenic amine reduction in the food treated by one of the physical intervention processes. In the report, γ-irradiation of raw materials with doses of 5, 10, and 15 kGy significantly reduced the contents of histamine, putrescine, tryptamine and spermidine by approximately 20–50% (but not tyramine, β-phenylethylamine, cadaverine, spermine and agmatine) in the final product of a fermented soybean food, viz., probably Doenjang [59]. However, it needs here to be noted that the irradiation even with the lowest dose resulted in an immediate and significant decrease in the numbers of *Bacillus* spp. and lactic acid bacteria, known as dominant bacteria in the food, by up to about 3 log CFU/g and 2 log CFU/g, respectively. As is well known, many of the physical intervention processes prevent the growth of fermenting microorganisms, as well as of biogenic amine-producing microorganisms, which may in turn not only delay fermentation, but also lead to abnormal fermentation caused by undesirable microorganisms resistant to the treatments [82]. Thus, introducing the processes would be somewhat challenging in the case of fermented soybean foods, considering the presence of fermenting and/or beneficial bioactive microorganisms in the foods.

The use of starter cultures has been suggested to be a successful way to enhance not only the quality and safety, but also the healthy functions, of fermented foods, causing less adverse organoleptic and unhealthy alterations [83,84,85]. Thus, with that a variety of microorganisms have been compared and screened for the ability to degrade biogenic amines and/or inability to produce biogenic amines in fermented foods, not only at the level of genus, species, or both, but also at the level of individual strain [73,74,75,76]. As for the fermentation of soybean, *Bacillus* strains have been steadily proposed as starter cultures to improve the sensory quality, but not the safety of fermented soybean foods [86,87]. On the contrary, less attention has been given to starter cultures for preventing or reducing biogenic amine formation in fermented soybean foods. As mentioned above, *Bacillus* spp. have been known as fermenting (or contaminating) microorganisms responsible for biogenic amine formation in different types of fermented soybean foods. Therefore, it is imperative to screen proper starter cultures (particularly *Bacillus* starter culture) with no or less ability to produce biogenic amines for the production of fermented soybean foods [62]. With respect to this, there have been a few reports in literature in which Doenjang and Cheonggukjang samples prepared with *B. subtilis* and *B. licheniformis* starter cultures, respectively, with low abilities to produce biogenic amines (the data on individual strains were not presented in the reports) contained lower levels of biogenic amines than those of previous studies [61,62]. Alternatively, the use of starter cultures that can degrade biogenic amines may facilitate the reduction of biogenic amines in fermented soybean foods [77]. At present, only two reports of biogenic amine-degrading starter cultures for the production of fermented soybean foods are available in literature as described below. In one study, *B. subtilis* and *B. amyloliquefaciens* strains which had been isolated from traditionally fermented soybean products degraded significant amounts of histamine (up to 71% of its initial concentration by *B. amyloliquefaciens*), tyramine (up to 70% by *B. amyloliquefaciens*), putrescine (up to 92% by *B. subtilis*) and cadaverine (up to 93% by *B. subtilis*) in cooked soybean after 10 days of fermentation [60]. In another study, *B. subtilis* and *B. amyloliquefaciens* strains which had been isolated from commercial fermented soybean products degraded 30–40% of tyramine in a phosphate buffer and probably thereby reduced tyramine content by 40–65% in the final product of Cheonggukjang, as compared to the control [63]. In addition, *B. subtilis* and *B. idriensis* strains isolated from a traditional fermented soybean food have been reported to be not only capable of degrading of, but also incapable of producing histamine and tyramine in vitro (but not applied to practical fermentation of soybean in the study) [88]. Consequently, it is feasible to screen *Bacillus* strains capable of degrading and/or incapable (or less capable) of producing biogenic amines, which would, in turn, make it possible to use them as starter cultures for reducing biogenic amine contents in fermented soybean foods. In the meantime, it is also necessary to fully identify and characterize *Bacillus* genes involved in the formation and degradation of biogenic amines, which would be helpful not only in selecting starter culture candidates but also in providing strategies to efficiently regulate the expression of these genes encoding relevant enzymes. Such molecular genetic studies would be further needed for better understanding of mechanisms by which intervention methods influencing intrinsic and extrinsic factors and/or microbial growth inhibit biogenic amine formation, at the level of gene. It is noteworthy that in addition to the aforementioned *Bacillus* starter cultures, strains of *E. faecium* and *L. plantarum* have also been proposed as starter cultures for fermented soybean foods because of their abilities to produce bacteriocin or to degrade biogenic amines, respectively [46,89]. Considering that the species are present as contaminants at relatively low levels in fermented soybean foods, as described above, further research is required prior to practical application to the fermentation of soybean in food industry.

Aside from the use of starter cultures, the production of biogenic amines has been known to be dependent on intrinsic and extrinsic factors of foods [77,78,79]. Furthermore, the factors may provide combined effects, especially in connection with technology applied, viz., the chemical and physical intervention measures described above [90]. As of now, however, the alterations of temperature, pH, a_w_, and Eh (as another important, but classical, biological intervention strategy), seem to be less preferable for studies on the reduction of biogenic amines in fermented soybean foods than other alternatives, considering that there are no relevant reports available, which might be because of the need to consider strict demands of consumers and governments on unique sensory properties and manufacturing processes of fermented soybean foods. Nonetheless, it is expected that the changes of the intrinsic and extrinsic factors within narrow ranges would be applicable, depending on the types of fermented soybean foods, if organoleptic evaluation is preceded. The intrinsic and extrinsic factors influencing biogenic amine formation in foods have been well reviewed in a recent article [8]. Biogenic amine reduction strategies, including chemical, physical and biological intervention methods, are summarized in Table 5.

## 7. Conclusions

The presence of histamine in fish is of concern in many countries due to its toxic potential and implications. Accordingly, there are specific legislations regarding the histamine content in fish and fish products in US, EU, and other countries. In contrast, the significance of biogenic amines in fermented soybean foods has been overlooked despite the presence not only of abundant precursor amino acids of biogenic amines in soybean, but also of microorganisms capable of producing biogenic amines during the fermentation of soybean. Fortunately, the studies published to date indicate that the amounts of biogenic amines in most fermented soybean food products are within the safe levels for human consumption. However, it should be pointed out that the contents of vasoactive biogenic amines in certain types and/or batches of fermented soybean food products are greater than toxic levels. Nonetheless, lack of both legislation and guidelines on the contents of biogenic amines in fermented soybean food products may lead to serious (or unnecessary) concerns about the safety of the fermented foods. Therefore, it is required to establish guidance levels of biogenic amines in fermented soybean food products based on information about the national daily intake of the fermented foods per person and the amounts of biogenic amines in different types of fermented soybean foods commonly consumed in each country.

Meanwhile, many efforts have been made to reduce biogenic amines in various fermented foods, particularly fermented sausage and cheese, whereas less attention has given to biogenic amines in fermented soybean foods. Consequently, there is at present a little information available regarding intervention methods to reduce biogenic amines in fermented soybean foods. Although empirical data on controlling biogenic amines in fermented soybean foods are not much in literature, several reports have suggested that the use of starter cultures capable of degrading and/or incapable of producing biogenic amines is a preferable way to biocontrol biogenic amines in fermented soybean foods because it probably causes less adverse organoleptic and unhealthy alterations as well as little changes in bacterial communities in the foods. Alterations of intrinsic and extrinsic factors, such as temperature, pH, a_w_, and Eh, in fermentation and manufacturing processes are also needed to be taken into consideration when biocontrol strategy is employed. With a successful reduction of biogenic amines in addition to significant health benefits, consumers may place a much higher value on fermented soybean foods.

## Figures and Tables

**Table 1:** Legal limits and toxic levels set by agencies for biogenic amines in food products.

Agency	Food	Toxicity Classification	Biogenic Amines (mg/kg) ^1^			Governing Entity	Ref.
			PHE	HIS	TYR		
Government	Fish ^2^ and fish products	Defect action level		50		United States	[17]
		Toxicity level		500			
		Maximum allowable level		200		Australia and New Zealand	[22]
		Maximum allowable level		200		Korea	[23]
		Maximum allowable level		400		China	[24]
	Fish ^3^ and fish products	Maximum allowable level		200			
International organization	Fresh fish ^4^	Defect action level		100		Europe	[15]
		Maximum allowable level		200			
	Enzymaticallyripened fish products ^4^	Defect action level		200			
		Maximum allowable level		400			
	Fish ^2^	Regulatory limit		100			[18]
	Fish sauce ^5^	Regulatory limit		400			
	Fish ^2^ and fish products	Maximum allowable level		200			[20]
		Decomposition indicator		100			[21]
		Hygiene and handling indicator		200			
	Fish sauce ^6^	Hygiene and handling indicator		400			
Independent research	General foods	Toxicity threshold	30	100	100–800		[6]
	Fish ^2^	Safe for consumption		<50			[16]
		Possibly toxic		50–200			
		Probably toxic		200–1000			
		Toxic and unsafe for human consumption		>1000			

^1^ PHE: β-phenylethylamine, HIS: histamine, TYR: tyramine; ^2^ *Scombridae*, *Clupeidae*, *Engraulidae*, *Pomatomide*, *Scombresosidae* and other fish species well known for high histamine content; ^3^ fish species without high histamine content; ^4^ *Scombridae* and *Clupeidae* families only; ^5^ produced by fermentation of fishery products; ^6^ prepared from fresh fish.

**Table 2:** Biogenic amine content in fermented soybean food products.

Fermented Soybean Products	N ^1^	Biogenic Amines (mg/kg) ^2^								Ref.
		TRP	PHE	PUT	CAD	HIS	TYR	SPD	SPM	
Cheonggukjang	7	6.7–236.4 ^3^	ND–40.8	4.7–121.3	2.1–20.2	1.3–54.3	0.7–483.1	39.6–59.2	7.1–14.7	[28]
	102	NT ^4^	NT	NT	NT	ND ^5^–755.40	ND–1913.51	NT	NT	[30]
	13	NT	NT	NT	NT	NT	117.5–2539.0	NT	NT	[33]
Chunjang	4	13.3–19.9	2.2–11.8	9.2–11.7	1.7–6.6	11.6–22.4	29.7–54.6	1.4–12.8	ND–2.9	[28]
	4	19.57–31.35	ND–6.79	3.26–28.59	ND–2.04	1.85–272.55	19.78–131.27	0.24–11.63	ND–1.49	[25]
Doenjang	14	6.1–234.1	ND–529.2	9.9–1453.7	0.3–65.4	1.5–952.0	3.4–1430.7	4.2–23.4	ND–10.2	[28]
	10	ND–449.8	ND–544.0	28.8–1076.6	2.7–144.1	1.4–329.2	12.5–967.6	ND–30.3	ND–9.8	[32]
	23	ND–2808.1	ND–8704.6	ND–4292.3	ND–3235.5	ND–2794.8	ND–6616.1	ND–8804.0	ND–9729.5	[34]
	7	13.5–45.9	3.3–65.0	46.7–168.2	ND–12.9	71.1–382.4	46.4–1190.7	ND–24.7	NT	[29]
Doubanjiang	7	ND–62.43	1.43–185.61	1.15–129.17	ND–0.17	ND	ND–25.75	ND–0.18	ND–1.69	[26]
Douchi	26	ND–440	ND–239	ND–596	ND–191	ND–808	ND–529	ND–719	ND–242	[36]
Gochujang	5	17.9–36.6	0.7–9.1	2.5–3.2	ND–1.1	0.6–1.3	2.1–4.9	1.6–3.4	1.4–1.8	[28]
	7	ND–8.1	1.5–24.8	10.4–36.4	ND–18.1	2.2–59.0	2.9–126.8	ND–14.5	NT	[29]
Miso	5	21.6–23.7	0.7–8.1	16.4–23.2	2.8–3.2	0.8–1.1	2.0–95.3	9.5–21.9	1.3–3.1	[28]
	40	ND–762	ND	ND–12	ND–201	ND–221	ND–49	ND	ND–216	[31]
	22	ND–9.71	2.38–11.76	2.69–14.09	ND–1.31	ND–24.42	ND–66.66	ND–28.31	ND–2.85	[27]
Natto	39	ND–301.0	ND	ND–27.0	ND–42.0	ND–457.0	ND–45.0	ND–124.0	ND–71.0	[35]
	21	ND–45.80	ND–51.50	ND–43.10	ND–36.80	ND–34.40	ND–300.20	246.50–478.10	18.80–80.10	[37]
Soy sauce	11	ND–45.8	1.5–121.6	2.5–1007.5	0.7–32.3	3.9–398.8	26.8–794.3	1.5–53.1	ND–16.1	[28]

^1^ Quantity of samples examined; ^2^ TRP: tryptamine, PHE: β-phenylethylamine, PUT: putrescine, CAD: cadaverine, HIS: histamine, TYR: tyramine, SPD: spermidine, SPM: spermine; ^3^ the range from minimum to maximum (the same number of digits is used after the decimal point in the values, as was presented in the corresponding references); ^4^ NT: not tested; ^5^ ND: not detected.

**Table 3:** Production of biogenic amines by bacteria isolated from fermented soybean food products.

Fermented Soybean Products	Isolates	N ^1^	Biogenic Amines (μg/mL) ^2^								Ref.
			TRP	PHE	PUT	CAD	HIS	TYR	SPD	SPM	
Chunjang	*Bacillus* spp. ^3^	89	0.45 ± 0.32 ^4^	0.85 ± 0.23	0.95 ± 0.55	ND ^5^	1.34 ± 1.19	1.41 ± 0.32	9.26 ± 5.73	2.17 ± 1.09	[25]
Doubanjiang	*Bacillus subtilis*	18	0.20 ± 0.45	0.67 ± 1.42	3.45 ± 1.29	1.03 ± 0.46	0.22 ± 0.65	0.59 ± 0.65	0.40 ± 0.20	1.29 ± 0.86	[26]
Douchi	*Bacillus subtilis*	4	NT ^6^	2.3 ± 4.5	NT	0.5 ± 0.6	18.7 ± 9.3	0.3 ± 0.5	NT	4.5 ± 5.2	[36]
	*Staphylococcus pasteuri*	1	NT	ND	NT	1.2	20.0	ND	NT	ND	
	*Staphylococcus capitis*	3	NT	5.4 ± 9.3	NT	1.1 ± 0.9	375.3 ± 197.0	1.1 ± 1.9	NT	2.7 ± 3.4	
Miso	*Staphylococcus pasteuri*	1	NT	6.4	ND	ND	28.1	NT	ND	NT	[31]
	*Bacillus* sp.	1	NT	2.7	1.6	2.1	15.3	NT	8.6	NT	
	*Bacillus amyloliquefaciens*	2	NT	1.6 ± 2.2	ND	1.8 ± 1.1	16.5 ± 8.6	NT	4.2 ± 5.9	NT	
	*Bacillus subtilis*	2	NT	ND	0.5 ± 0.7	0.6 ± 0.8	29.9 ± 13.4	NT	5.0 ± 7.1	NT	
	*Bacillus megaterium*	2	NT	7.7 ± 0.4	ND	ND	14.6 ± 2.8	NT	4.7 ± 6.6	NT	
Natto	*Bacillus subtilis*	80	6.17 ± 3.98	11.2 ± 9.17	7.59 ± 3.06	0.94 ± 1.67	9.91 ± 1.61	30.6 ± 21.7	3.34 ± 1.82	27.2 ± 12.7	[37]
	*Bacillus subtilis*	2	NT	2.4 ± 3.3	NT	1.5 ± 0.1	15.5 ± 2.9	NT	NT	NT	[35]
	*Staphylococcus pasteuri*	2	NT	ND	NT	1.1 ± 0.1	15.0 ± 1.3	NT	NT	NT	

^1^ Quantity of bacterial samples examined; ^2^ TRP: tryptamine, PHE: β-phenylethylamine, PUT: putrescine, CAD: cadaverine, HIS: histamine, TYR: tyramine, SPD: spermidine, SPM: spermine; ^3^ *Bacillus* spp. were identified to be *B. subtilis* (91.0%), *B. coagulans* (4.5%), *B. licheniformis* (1.1%) and *B. firmus* (1.1%); ^4^ mean ± standard deviation (the same number of digits is used after the decimal point in the values, as was presented in the corresponding references); ^5^ ND: not detected; ^6^ NT: not tested.

**Table 4:** Genes encoding amino acid decarboxylases in *Bacillus* spp. and *Enterococcus* spp. registered in the NCBI database.

Species	Strain ^1^	Source	Gene for Amino Acid Decarboxylase ^2^	No. of Amino Acids	Locus Name	Accession (Version)	Size (bp)
*B. subtilis*	*B. subtilis* subsp. *subtilis* strain 168	Isolated strain	*odc*-Az	331	BACYACA	L77246.1	996
			*ldc*	490	AF012285	AF012285.1	1473
*B. licheniformis*/*B. coagulans*	*B. licheniformis* A5/*B. coagulans* SL5	Isolated strain	*hdc*	146	AB553282/AB553281	AB553282.1/AB553281.1	441
*E. faecium*	*E. faecium* strain 993	Isolated strains	*odc*	235	PDLZ01000281	PDLZ01000281.1	707
	*E. faecium* ATCC 700221	ATCC	*ldc*	191	CP014449	CP014449.1	576
	*E. faecium* ATCC 700221	ATCC	*tdc*	611	CP014449	CP014449.1	1836
*E. faecalis*	*E. faecalis* ATCC 51299	ATCC	*ldc*	194	JSES01000022	JSES01000022.1	585
	*E. faecalis* ATCC 19433	Type strain	*tdc*	620	KB944589	KB944589.1	1863

^1^ Genes found in a single strain of each *Bacillus* species have been registered, while those of *Enterococcus* spp. found in multiple strains have been separately assigned to different loci, of which a representative locus is presented in the table; ^2^ *odc*-Az: 37.0% identity over 119 amino acids to the *E. coli* ornithine decarboxylase antizyme, *odc*: gene for ornithine decarboxylase, *ldc*: gene for lysine decarboxylase, *hdc*: gene for histidine decarboxylase, *tdc*: gene for tyrosine decarboxylase; ATCC: the American Type Culture Collection.

**Table 5:** Biogenic amine reduction strategies for food products.

Parameter Categories		Highly Effective Strategies
Chemical intervention		Nicotinic acid [43], glycine [64], garlic [65], clove [65], clove and sodium chloride [66], clove with potassium sorbate and sodium benzoate [67]
Physical intervention		Irradiation [59,68], high hydrostatic pressure [69,70], modified atmospheric packaging and temperature [71,72]
Biological intervention	Starter cultures	Lactic acid bacteria [84], *Lactobacillus sake* + *Pediococcus pentosaceus* + *Staphylococcus carnosus* + *S. xylosus* [73], *S. carnosus* [74], *S. xylosus* [74,75,76], *L. plantarum* [89], *Bacillus subtilis* [60,63,88], *B. amyloliquefaciens* [60,63], *B. licheniformis* [62], *B. idriensis* [88], *B. subtilis* + *Aspergillus oryzae* + *Mucor racemosus* [61]
	Intrinsic and extrinsic factors	Temperature, pH, a_w_, Eh [77,78,79]

## References

[1] Joosten H.M.L.J., Nuñez M. (1996). Prevention of histamine formation in cheese by bacteriocin-producing lactic acid bacteria. Appl. Environ. Microbiol..

[2] Taylor S.L., Guthertz L.S., Leatherwood H., Tillman F., Lieber E.R. (1978). Histamine production by foodborne bacterial species. J. Food Saf..

[3] Shin D., Jeong D. (2015). Korean traditional fermented soybean products: *Jang*. J. Ethn. Foods.

[4] Toro-Funes N., Bosch-Fuste J., Latorre-Moratalla M.L., Veciana-Nogués M.T., Vidal-Carou M.C. (2015). Biologically active amines in fermented and non-fermented commercial soybean products from the Spanish market. Food Chem..

[5] Mah J.-H. (2015). Fermented soybean foods: Significance of biogenic amines. Austin J. Nutr. Food Sci..

[6] Brink B., Damirik C., Joosten H.M.L.J., Huis In’t Veld J.H.J. (1990). Occurrence and formation of biologically active amines in foods. Int. J. Food Microbiol..

[7] Halász A., Baráth Á., Simon-Sarkadi L., Holzapfel W. (1994). Biogenic amines and their production by microorganisms in food. Trends Food Sci. Technol..

[8] Gardini F., Özogul Y., Suzzi G., Tabanelli G., Özogul F. (2016). Technological factors affecting biogenic amine content in foods: A review. Front. Microbiol..

[9] Stratton J.E., Hutkins R.W., Taylor S.L. (1991). Biogenic amines in cheese and other fermented foods: A review. J. Food Prot..

[10] Rice S.L., Eitenmiller R.R., Koehler P.E. (1976). Biologically active amines in foods: A review. J. Milk Food Technol..

[11] Fernández-Reina A., Urdiales J., Sánchez-Jiménez F. (2018). What we know and what we need to know about aromatic and cationic biogenic amines in the gastrointestinal tract. Foods.

[12] Barbieri F., Montanari C., Gardini F., Tabanelli G. (2019). Biogenic amine production by lactic acid bacteria: A Review. Foods.

[13] Byun B.Y., Bai X., Mah J.-H. (2013). Bacterial contribution to histamine and other biogenic amine content in *Juk* (Korean traditional congee) cooked with seafood. Food Sci. Biotechnol..

[14] U.S. Food and Drug Administration (1982). Defect action levels for histamine in tuna: Availability of guide. Fed. Reg..

[15] European Economic Commission (EEC) (1991). Council Directive 91/493/EEC of 22 July 1991 laying down the health conditions for the production and the placing on the market of fishery products. Off. J. Eur. Comm..

[16] Shalaby A.R. (1996). Significance of biogenic amines to food safety and human health. Food Res. Int..

[17] U.S. Food and Drug Administration (FDA) (2011). Fish and Fishery Products Hazards and Controls Guidance.

[18] European Commision (EC) (2005). Commission Regulation No. 2073/2005 of 15th November 2005 on microbiological criteria for Foodstuffs. Off. J. Eur. Union.

[19] European Food Safety Authority (EFSA) (2011). Scientific opinion on risk based control of biogenic amine formation in fermented foods. EFSA J..

[20] Food and Agriculture Organization of the United Nations/World Health Organization (FAO/WHO) (2013). Joint FAO/WHO Expert Meeting on the Public Health Risk of Histamine and Other Biogenic Amines from Fish and Fishery Products.

[21] Codex (2012). Discussion paper histamine. Joint FAO/WHO Food Standards Programme, Codex Committee on Fish and Fishery Products, Thirty-Second Session (CX/FFP 12/32/14), Bali, Indonesia, 1–5 October 2012.

[22] Food Standards Australia New Zealand (FSANZ) (2016). Imported Food Risk Statement Fish and Fish Products from the Families Specifies and Histamine.

[23] The Ministry of Food and Drug Safety (MFDS) (2017). Food Code, Notification No. 2017-57.

[24] China National Standards (2016). GB 2733-2015 National Food Safety Standards for Fresh and Frozen Animal Aquatic Products.

[25] Bai X., Byun B.Y., Mah J.-H. (2013). Formation and destruction of biogenic amines in *Chunjang* (a black soybean paste) and *Jajang* (a black soybean sauce). Food Chem..

[26] Byun B.Y., Bai X., Mah J.-H. (2013). Occurrence of biogenic amines in *Doubanjiang* and *Tofu*. Food Sci. Biotechnol..

[27] Byun B.Y., Mah J.-H. (2012). Occurrence of biogenic amines in *Miso*, Japanese traditional fermented soybean paste. J. Food Sci..

[28] Cho T.-Y., Han G.-H., Bahn K.-N., Son Y.-W., Jang M.-R., Lee C.-H., Kim S.-H., Kim D.-B., Kim S.-B. (2006). Evaluation of biogenic amines in Korean commercial fermented foods. Korean J. Food Sci. Technol..

[29] Kim T.-K., Lee J.-I., Kim J.-H., Mah J.-H., Hwang H.-J., Kim Y.-W. (2011). Comparison of ELISA and HPLC methods for the determination of biogenic amines in commercial *Doenjang* and *Gochujang*. Food Sci. Biotechnol..

[30] Ko Y.-J., Son Y.-H., Kim E.-J., Seol H.-G., Lee G.-R., Kim D.-H., Ryu C.-H. (2012). Quality properties of commercial *Chungkukjang* in Korea. J. Agric. Life Sci..

[31] Kung H.-F., Tsai Y.-H., Wei C.-I. (2007). Histamine and other biogenic amines and histamine-forming bacteria in miso products. Food Chem..

[32] Lee H.T., Kim J.H., Lee S.S. (2009). Analysis of microbiological contamination and biogenic amines content in traditional and commercial Doenjang. J. Food Hyg. Saf..

[33] Oh S.-J., Mah J.-H., Kim J.-H., Kim Y.-W., Hwang H.-J. (2012). Reduction of tyramine by addition of *Schizandra chinensis* Baillon in Cheonggukjang. J. Med. Food.

[34] Shukla S., Park H.-K., Kim J.-K., Kim M. (2010). Determination of biogenic amines in Korean traditional fermented soybean paste (Doenjang). Food Chem. Toxicol..

[35] Tsai Y.-H., Chang S.-C., Kung H.-F. (2007). Histamine contents and histamine-forming bacteria in natto products in Taiwan. Food Control.

[36] Tsai Y.-H., Kung H.-F., Chang S.-C., Lee T.-M., Wei C.-I. (2007). Histamine formation by histamine-forming bacteria in douchi, a Chinese traditional fermented soybean product. Food Chem..

[37] Kim B., Byun B.Y., Mah J.-H. (2012). Biogenic amine formation and bacterial contribution in *Natto* products. Food Chem..

[38] Shukla S., Kim J.-K., Kim M., El-Shemy H. (2011). Occurrence of biogenic amines in soybean food products. Soybean and Health.

[39] Onda T., Yanagida F., Shinohara T., Yokotsuka K. (2003). Time series analysis of aerobic bacterial flora during Miso fermentation. Lett. Appl. Microbiol..

[40] Tamang J.P., Tamang J.P., Kailasapathy K. (2010). Diversity of fermented foods. Fermented Foods and Beverages of the World.

[41] Ibe A., Nishima T., Kasai N. (1992). Formation of tyramine and histamine during soybean paste (Miso) fermentation. Jpn. J. Toxicol. Environ. Health.

[42] Ibe A., Nishima T., Kasai N. (1992). Bacteriological properties of and amine-production conditions for tyramine-and histamine-producing bacterial strains isolated from soybean paste (Miso) starting materials. Jpn. J. Toxicol. Environ. Health.

[43] Kang H.-R., Kim H.-S., Mah J.-H., Kim Y.-W., Hwang H.-J. (2018). Tyramine reduction by tyrosine decarboxylase inhibitor in *Enterococcus faecium* for tyramine controlled *cheonggukjang*. Food Sci. Biotechnol..

[44] Oh E.J., Oh M.-H., Lee J.M., Cho M.S., Oh S.S. (2008). Characterization of microorganisms in *Eoyukjang*. Korean J. Food Sci. Technol..

[45] Sarkar P., Tamang J.P., Cook P.E., Owens J. (1994). Kinema-a traditional soybean fermented food: Proximate composition and microflora. Food Microbiol..

[46] Yoon M.Y., Kim Y.J., Hwang H.-J. (2008). Properties and safety aspects of *Enterococcus faecium* strains isolated from *Chungkukjang*, a fermented soy product. LWT-Food Sci. Technol..

[47] Coton E., Coton M. (2005). Multiplex PCR for colony direct detection of Gram-positive histamine-and tyramine-producing bacteria. J. Microbiol. Methods.

[48] Komprda T., Sládková P., Petirová E., Dohnal V., Burdychová R. (2010). Tyrosine-and histidine-decarboxylase positive lactic acid bacteria and enterococci in dry fermented sausages. Meat sci..

[49] Jeon A.R., Lee J.H., Mah J.-H. (2018). Biogenic amine formation and bacterial contribution in *Cheonggukjang*, a Korean traditional fermented soybean food. LWT-Food Sci. Technol..

[50] Caruso M., Fiore C., Contursi M., Salzano G., Paparella A., Romano P. (2002). Formation of biogenic amines as criteria for the selection of wine yeasts. World J. Microbiol. Biotechnol..

[51] Izquierdo-Pulido M., Font-Fábregas J., Vidal-Carou C. (1995). Influence of *Saccharomyces cerevisiae* var. *uvarum* on histamine and tyramine formation during beer fermentation. Food Chem..

[52] Nout M.K.R., Ruiker M.M.W., Bouwmeester H.M., Beljaars P.R. (1993). Effect of processing conditions on the formation of biogenic amines and ethyl carbamate in soybean tempe. J. Food Saf..

[53] Fuell C., Elliott K.A., Hanfrey C.C., Franceschetti M., Michael A.J. (2010). Polyamine biosynthetic diversity in plants and algae. Plant Physiol. Biochem..

[54] Kumar A., Taylor M.A., Arif S.A.M., Davies H.V. (1996). Potato plants expressing antisense and sense S-adenosylmethionine decarboxylase (SAMDC) transgenes show altered levels of polyamines and ethylene: Antisense plants display abnormal phenotypes. Plant J..

[55] Kalaè P., Krausová P. (2005). A review of dietary polyamines: Formation, implications for growth, and health and occurrence in foods. Food Chem..

[56] Liu Z.-F., Wei Y.-X., Zhang J.-J., Liu D.-H., Hu Y.-Q., Ye X.-Q. (2011). Changes in biogenic amines during the conventional production of stinky tofu. Int. J. Food Sci. Technol..

[57] Woolridge D.P., Vazquez-Laslop N., Markham P.N., Chevalier M.S., Gerner E.W., Neyfakh A.A. (1997). Efflux of the natural polyamine spermidine facilitated by the *Bacillus subtilis* multidrug transporter Blt. J. Biol. Chem..

[58] Righetti L., Tassoni A., Bagni N. (2008). Polyamines content in plant derived food: A comparison between soybean and Jerusalem artichoke. Food Chem..

[59] Kim J.-H., Ahn H.-J., Kim D.-H., Jo C., Yook H.-S., Park H.-J., Byun M.-W. (2003). Irradiation effects on biogenic amines in Korean fermented soybean paste during fermentation. J. Food Sci..

[60] Kim Y.S., Cho S.H., Jeong D.Y., Uhm T.-B. (2012). Isolation of biogenic amines-degrading strains of *Bacillus subtilis* and *Bacillus amyloliquefaciens* from traditionally fermented soybean products. Korean J. Microbiol..

[61] Shukla S., Lee J.S., Park H.-K., Yoo J.-A., Hong S.-Y., Kim J.-K., Kim M. (2015). Effect of novel starter culture on reduction of biogenic amines, quality improvement, and sensory properties of *Doenjang*, a traditional Korean soybean fermented sauce variety. J. Food Sci..

[62] Kim S.-Y., Kim H.-E., Kim Y.-S. (2017). The potentials of *Bacillus licheniformis* strains for inhibition of *B. cereus* growth and reduction of biogenic amines in *cheonggukjang* (Korean fermented unsalted soybean paste). Food Control.

[63] Kang H.-R., Lee Y.-L., Hwang H.-J. (2017). Potential for application as a starter culture of tyramine-reducing strain. J. Korean Soc. Food Sci. Nutr..

[64] Mah J.-H., Hwang H.-J. (2009). Effects of food additives on biogenic amine formation in *Myeolchi-jeot*, a salted and fermented anchovy (*Engraulis japonicus*). Food Chem..

[65] Mah J.-H., Kim Y.J., Hwang H.-J. (2009). Inhibitory effects of garlic and other spices on biogenic amine production in *Myeolchi-jeot*, Korean salted and fermented anchovy product. Food Control.

[66] Wendakoon C.N., Sakaguchi M. (1993). Combined effect of sodium chloride and clove on growth and biogenic amine formation of *Enterobacter aerogenes* in mackerel muscle extract. J. Food Prot..

[67] Wendakoon C.N., Sakaguchi M. (1993). Combined effects of cloves with potassium sorbate and sodium benzoate on the growth and biogenic amine production of *Enterobacter aerogenes*. Biosci. Biotechnol. Biochem..

[68] Rabie M.A., Siliha H., el-Saidy S., el-Badawy A.A., Malcata F.X. (2010). Effects of γ-irradiation upon biogenic amine formation in Egyptian ripened sausages during storage. Innov. Food Sci. Emerg. Technol..

[69] Novella-Rodriguez S., Veciana-Nogués M.T., Saldo J., Vidal-Carou M.C. (2002). Effects of high hydrostatic pressure treatments on biogenic amine contents in goat cheeses during ripening. J. Agric. Food Chem..

[70] Ruiz-Capillas C., Colmenero F.J., Carrascosa A.V., Muñoz R. (2007). Biogenic amine production in Spanish dry-cured “chorizo” sausage treated with high-pressure and kept in chilled storage. Meat Sci..

[71] Patsias A., Chouliara I., Paleologos E.K., Savvaidis I., Kontominas M.G. (2006). Relation of biogenic amines to microbial and sensory changes of precooked chicken meat stored aerobically and under modified atmosphere packaging at 4 °C. Eur. Food Res. Technol..

[72] Ruiz-Capillas C., Pintado T., Jiménez-Colmenero F. (2012). Biogenic amine formation in refrigerated fresh sausage “chorizo” keeps in modified atmosphere. J. Food Biochem..

[73] Ayhan K., Kolsarici N., Özkan G.A. (1999). The effects of a starter culture on the formation of biogenic amines in Turkish soudjoucks. Meat Sci..

[74] Bover-Cid S., Izquierdo-Pulido M., Vidal-Carou M.C. (1999). Effect of proteolytic starter cultures of *Staphylococcus* spp. on biogenic amine formation during the ripening of dry fermented sausages. Int. J. Food Microbiol..

[75] Gardini F., Martuscelli M., Crudele M.A., Pararella A., Suzzi G. (2002). Use of *Staphylococcus xylosus* as a starter culture in dried sausages: Effect on the biogenic amine content. Meat Sci..

[76] Mah J.-H., Hwang H.-J. (2009). Inhibition of biogenic amine formation in a salted and fermented anchovy by *Staphylococcus xylosus* as a protective culture. Food Control.

[77] Alvarez M.A., Moreno-Arribas M.V. (2014). The problem of biogenic amines in fermented foods and the use of potential biogenic amine-degrading microorganisms as a solution. Trends Food Sci. Technol..

[78] Naila A., Flint S., Fletcher G., Bremer P., Meerdink G. (2010). Control of biogenic amines in food-existing and emerging approaches. J. Food Sci..

[79] Stadnik J., Rai V.R., Bai J.A. (2014). Significance of biogenic amines in fermented foods and methods of their control. Beneficial Microbes in Fermented and Functional Foods.

[80] Carocho M., Barreiro M.F., Morales P., Ferreira I.C. (2014). Adding molecules to food, pros and cons: A review on synthetic and natural food additives. Compr. Rev. Food Sci. Food Saf..

[81] Floros J.D., Newsome R., Fisher W., Barbosa-Cánovas G.V., Chen H., Dunne C.P., German J.B., Hall R.L., Heldman D.R., Karwe M.V. (2010). Feeding the world today and tomorrow: The importance of food science and technology. Compr. Rev. Food Sci. Food Saf..

[82] Fang S.-H., Lai Y.-J., Chou C.-C. (2013). The susceptibility of *Streptococcus thermophilus* 14085 to organic acid, simulated gastric juice, bile salt and disinfectant as influenced by cold shock treatment. Food Microbiol..

[83] Hansen E.B. (2002). Commercial bacterial starter cultures for fermented foods of the future. Int. J. Food Microbiol..

[84] Leroy F., De Vuyst L. (2004). Lactic acid bacteria as functional starter cultures for the food fermentation industry. Trends Food Sci. Technol..

[85] Leroy F., Verluyten J., De Vuyst L. (2006). Functional meat starter cultures for improved sausage fermentation. Int. J. Food Microbiol..

[86] Omafuvbe B.O., Abiose S.H., Shonukan O.O. (2002). Fermentation of soybean (*Glycine max*) for soy-*daddawa* production by starter cultures of *Bacillus*. Food Microbiol..

[87] Tamang J.P., Nikkuni S. (1996). Selection of starter cultures for the production of kinema, a fermented soybean food of the Himalaya. World J. Microbiol. Biotechnol..

[88] Eom J.S., Seo B.Y., Choi H.S. (2015). Biogenic amine degradation by *Bacillus* species isolated from traditional fermented soybean food and detection of decarboxylase-related genes. J. Microbiol. Biotechnol..

[89] Yi-Chen L., Hsien-Feng K., Ya-Ling H., Chien-Hui W., Yu-Ru H., Yung-Hsiang T. (2016). Reduction of biogenic amines during miso fermentation by *Lactobacillus plantarum* as a starter culture. J. Food Prot..

[90] Ruiz-Capillas C., Herrero A.M. (2019). Impact of biogenic amines on food quality and safety. Foods.

